# Interdisciplinary Online Hackathons as an Approach to Combat the COVID-19 Pandemic: Case Study

**DOI:** 10.2196/25283

**Published:** 2021-02-08

**Authors:** Katarina Braune, Pablo-David Rojas, Joscha Hofferbert, Alvaro Valera Sosa, Anastasiya Lebedev, Felix Balzer, Sylvia Thun, Sascha Lieber, Valerie Kirchberger, Akira-Sebastian Poncette

**Affiliations:** 1 Department of Paediatric Endocrinology and Diabetes Charité – Universitätsmedizin Berlin, Corporate Member of Freie Universität Berlin Humboldt-Universität zu Berlin, and Berlin Institute of Health Berlin Germany; 2 Hacking Health Berlin Berlin Germany; 3 Berlin Institute of Health Berlin Germany; 4 CityLAB Berlin Building Health Lab Berlin Germany; 5 Department of Design and Typologies Technische Universität Berlin Berlin Germany; 6 Charité – Universitätsmedizin Berlin, Corporate Member of Freie Universität Berlin Humboldt-Universität zu Berlin, and Berlin Institute of Health Department of Anesthesiology and Intensive Care Medicine Berlin Germany; 7 Einstein Center Digital Future Berlin Germany; 8 Executive Board Charité – Universitätsmedizin Berlin, Corporate Member of Freie Universität Berlin Humboldt-Universität zu Berlin, and Berlin Institute of Health Berlin Germany

**Keywords:** hackathon, COVID-19, digital health, mentoring, interdisciplinarity, interoperability, SARS-CoV-2, public health, innovation, collaboration, hack, mentor, case study, online health care, challenge, implementation, plan, collaboration

## Abstract

**Background:**

The COVID-19 outbreak has affected the lives of millions of people by causing a dramatic impact on many health care systems and the global economy. This devastating pandemic has brought together communities across the globe to work on this issue in an unprecedented manner.

**Objective:**

This case study describes the steps and methods employed in the conduction of a remote online health hackathon centered on challenges posed by the COVID-19 pandemic. It aims to deliver a clear implementation road map for other organizations to follow.

**Methods:**

This 4-day hackathon was conducted in April 2020, based on six COVID-19–related challenges defined by frontline clinicians and researchers from various disciplines. An online survey was structured to assess: (1) individual experience satisfaction, (2) level of interprofessional skills exchange, (3) maturity of the projects realized, and (4) overall quality of the event. At the end of the event, participants were invited to take part in an online survey with 17 (+5 optional) items, including multiple-choice and open-ended questions that assessed their experience regarding the remote nature of the event and their individual project, interprofessional skills exchange, and their confidence in working on a digital health project before and after the hackathon. Mentors, who guided the participants through the event, also provided feedback to the organizers through an online survey.

**Results:**

A total of 48 participants and 52 mentors based in 8 different countries participated and developed 14 projects. A total of 75 mentorship video sessions were held. Participants reported increased confidence in starting a digital health venture or a research project after successfully participating in the hackathon, and stated that they were likely to continue working on their projects. Of the participants who provided feedback, 60% (n=18) would not have started their project without this particular hackathon and indicated that the hackathon encouraged and enabled them to progress faster, for example, by building interdisciplinary teams, gaining new insights and feedback provided by their mentors, and creating a functional prototype.

**Conclusions:**

This study provides insights into how online hackathons can contribute to solving the challenges and effects of a pandemic in several regions of the world. The online format fosters team diversity, increases cross-regional collaboration, and can be executed much faster and at lower costs compared to in-person events. Results on preparation, organization, and evaluation of this online hackathon are useful for other institutions and initiatives that are willing to introduce similar event formats in the fight against COVID-19.

## Introduction

The COVID-19 outbreak is first and foremost a human tragedy, affecting the health of millions of people and overwhelming health care systems and the global economy [1]. This devastating pandemic has brought together communities across the globe to tackle this issue in an unprecedented manner.

Hackathons are collaborative multiday events that bring people from different professional and personal backgrounds face to face in a venue to work on specific challenges. Previous publications have shown how health hackathons work as an effective model of interdisciplinary collaboration, capable of forwarding recommendations and viable solutions effectively [2-36]; however, they often require several months of preparation and considerable financing [6,34].

Little is so far known about how health hackathons can be executed remotely and in quick response to ongoing circumstances, as well as how they can contribute to solving challenges related to the COVID-19 pandemic, considering the amount and fast pace of evidence gathered on its impact and possible solutions [37-48].

The following case study describes the execution of an online hackathon dedicated to the challenges of the COVID-19 pandemic and participant experience, which will be useful to other organizations and institutions in the health care sector.

## Methods

### Study Setting

Hacking Health is a global nonprofit organization that pairs innovators with health care experts [6]. Its Berlin chapter organized its first online hackathon in partnership with Charité – Universitätsmedizin Berlin (the largest university hospital in Europe), the Berlin Institute of Health, Diabetes Center Berne, and Data Natives (an online community of data scientists and developers). The event, called EasterHack [49], ran from Friday evening to Monday afternoon over the Easter weekend in 2020. Participants included physicians, health care professionals, researchers, patients, entrepreneurs, engineers, designers, developers, etc. Participation was open to everyone worldwide and was free of charge.

### Challenges

The most critical issues caused by, or associated with, COVID-19 and the current state of possible solutions were reviewed and discussed in a series of five virtual meetings held across the interdisciplinary Hacking Health Berlin community in cooperation with the hospital’s executive board and different departments of Charité – Universitätsmedizin Berlin.

A summary of this review was sent to, and discussed with, frontline clinicians and researchers for consideration, to ponder on its relevance, and to raise other questions related to the most pressing issues in their line of work. It was highly relevant for identifying unmet needs and urgent problems among health care professionals, vulnerable patient groups, and more generally on a population level, rather than ideas for solutions. Thereafter, a total of six challenges were identified by the Hacking Health Berlin team (Table 1) in close cooperation with the university hospital’s executive board and other institutes and departments (Institute of Hygiene and Tropical Medicine, Department of Anesthesiology and Intensive Care Medicine, Department of Psychosomatic Medicine, Department of Paediatric Endocrinology and Diabetes of Charité – Universitätsmedizin Berlin) and the open-source initiative CoEpi.org, an international online community of volunteers with different skillsets working on open-source solutions for privacy-first contact tracing. Participants were allowed to use their choice of technology to solve these challenges, without constraint or limitation.

**Table 1:** Overview of the hackathon’s challenges. A total of six COVID-19–related challenges were identified in close cooperation with frontline physicians and researchers.

Challenge	Questions/considerations
1. Protect high-risk populations	How can we better support the medical needs of high-risk populations (eg, people with pre-existing conditions such as cancer, diabetes, and immunodeficiencies)? How can we identify high-risk patients and isolate them from the general population? How can we support high-risk patients with preventive ambulatory treatment? How can we deliver care for people with chronic conditions during a pandemic?
2. Protect health care workers	How can we prevent medical staff from getting infected with SARS-CoV-2 in health care facilities? How can we manage the increasing demand for personal protective equipment in patient care?
3. Privacy-first contact tracing and digital epidemiology	What can we build on top of open-source solutions (such as CoEpi.org and COVID-watch.org) to allow users to opt in to disclose anonymous, nonidentifiable information that public health officials would find useful? How can we authenticate positive COVID-19 tests without requiring public health authorities to do extra work to authenticate them for us? How can we collect more information on Bluetooth low-energy proximity contact event duration and distance to inform risk scores? How can we automate the provisional diagnosis of COVID-19 based solely on self-reported symptoms and self-collected data?
4. Improve intensive care	How can mechanical ventilation be improved to reduce long-term damage to the lungs? How can we ease prone positioning in ventilated patients? How can we reduce the formation of aerosols? How can we visually monitor agitated and hyperactive patients in the ICU^a^ to prevent them from extubating or hurting themselves? How can we give confused ICU patients in-room, time, and situation orientation? How can we make the ICU a more natural and pleasant environment? How can we apply evidence-based medicine in all ICUs by tele-ICU? How can we prevent long-term complications following ICU treatment such as controlled cortical impact and postintensive care syndrome?
5. Protect our mental health	How can we support health care workers in dealing with the stress and trauma that they might experience during a pandemic? How can we identify and offer support to people, families, friends, and/or coworkers who are running into mental health issues? How can we predict critical mental states through early detection?
6. Your idea	Find a novel solution for your individual COVID-19 challenge, if it has not been listed above

^a^ICU: intensive care unit.

### Preparation

The core organizing team consisted of 7 volunteers to set up digital tools; scheduling; marketing outreach strategy for recruitment of participants, cooperating partners, participant and mentor communication; and coordination. An implementation strategy previously designed and used for in-person hackathons by Charité – Universitätsmedizin Berlin and Hacking Health Berlin [6] was adjusted accordingly.

Due to the novelty and urgency of the topic, organization of the hackathon began only 2 weeks prior to the event being completely prepared and executed online, which is different from former hackathons. In April 2020, travel and assembly restrictions, as well as social distancing recommendations, did not allow in-person meetings in Europe. However, the online nature of the event allowed participants and mentors from all over the world to participate regardless of their location, avoiding traveling costs and time.

### Participant Engagement

Recent innovation studies have demonstrated interdisciplinarity among participants and within-team compositions are essential for the success of hackathon projects [50,51]. Therefore, it is essential to recruit participants from various relevant fields of expertise such as biomedical research, health care, psychology, education, design, economics, data science, computer science, engineering, social sciences, and politics. Future end-users of the solutions created at a hackathon should be included as early as possible to actively contribute to the cocreation process.

To attract and recruit a diverse and interdisciplinary group of participants, previously existing multidisciplinary networks on LinkedIn [52] and Slack [53] actively engaged in health care innovation, and cooperating with Hacking Health Berlin, were targeted. This provided a great opportunity to reach participants, mentors, and cooperation partners who supported organizers and participating teams in developing projects before, during, and after the hackathon. As one of the largest local networks of innovation in health care, Hacking Health Berlin was able to recruit a large number of interdisciplinary skilled participants within only 5 days. Professionals working in relevant fields and interested in digital health were also targeted through promotional campaigns using LinkedIn, Facebook, Meetup, and other social media channels.

Participants and mentors were able to sign up via an online form, where they provided information on their skillset, shared their experience level and accomplishments from previous hackathons, their motivations to participate, their desired team role during the hackathon, and a brief outline of their project idea.

### Online Tools

To prepare for the hackathon, the organizers used Airtable [54] to arrange schedules, responsibilities, marketing, registration procedures, jury criteria definition, and the collection and structuring of all information provided to the teams before, throughout, and after the event. The Hacking Health Berlin team and external co-organizers had access to this platform.

The online instant messenger tool Slack [53] was used for communication during the event to coordinate actions and share data via text and video. Public and closed subchannels were created for the organizing team, mentors, judges, and participants to communicate and execute activities. Additionally, participating teams created their private subchannels on Slack to communicate internally.

Mentoring sessions were coordinated and held on the Mentornity platform [55], as described in a subsequent point. For the opening and closing ceremonies, moderations, pitches, and prototype showcasing sessions, video conferences were held via Zoom and live streamed to the public on Facebook and YouTube.

### The Hackathon

**Figure 1:**
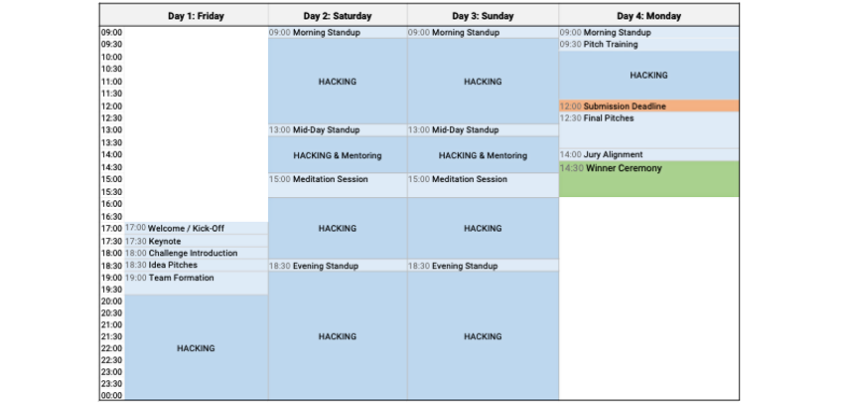
Activities and schedule of a 4-day online hackathon.

The hackathon was conducted over a period of 4 days, from Friday to Monday afternoon. An example schedule is shown in Figure 1.

A keynote from the director of the interoperability unit of the Berlin Institute of Health opened the event, motivated the teams, and pointed out the importance of this online hackathon to create solutions for the challenges of the COVID-19 pandemic.

Following the opening ceremony, individual participants and pre-existing teams pitched their ideas for the challenges they selected. Additional participants joined the team of their choice. Team composition is a crucial part of hackathons. As previous research shows, highly diverse and multidisciplinary teams are more likely to be effective in developing creative and sustainable solutions [50,51]. This is particularly relevant in the complex field of digital health, where experts from different fields and areas are needed for development such as health care workers, patients and caregivers, developers, engineers, data scientists, designers, and experts in economics, legal and regulatory affairs. Team building was supported by volunteers from the Hacking Health Berlin team to foster interdisciplinary and international cooperation. A maximum of 6 people per team was recommended.

After teams were formed, the idea and prototype were developed collaboratively over the course of 4 days aided by mentoring sessions. Daily morning and evening stand-up sessions were held virtually, open to all organizers, mentors, and participants.

On day 4, solutions and prototypes were presented to the public and evaluated by an interdisciplinary jury entitled to award the most elaborate and promising solutions based on the criteria described below.

### Mentoring

Mentors provided participants with valuable expert knowledge about medicine, design, business, IT (information technology), and other areas of expertise. They indicated their availability for mentoring within the 48-hour span of days 2 and 3. This setting was well embraced, especially by health care professionals—despite the pressing pandemic situation—and other professionals working from home.

One week prior to the hackathon event, mentors with previous mentoring experience at Hacking Health Berlin events were recruited via email. New mentors were recruited through various local and international networks with the use of social media campaigns and direct messaging via networking platforms. In both cases, mentors were asked to fill in an online form indicating fields of expertise and availability (ie, convenient time slots). This information was transferred to the online platform Mentornity. The platform showed each mentor’s profile and facilitated video call sessions for each team. Simultaneously, participants created team profiles for their project, searched mentors by expertise area, and booked at least one mandatory mentoring session made available by the mentor. The platform’s analysis feature tracked the number of mentoring sessions held by every mentor and team. Likewise, mentors were encouraged to sign up for Slack—the hackathon’s communication platform—to keep everyone informed on sessions prior to and during the event.

Two experts in the field of communication and public speaking offered dedicated pitch training to help teams develop focused final pitches that adhered to the 3-minute time limit.

### Jury and Project Evaluation

The interdisciplinary jury was composed of 10 members: a professor of anesthesiology and computer science, a pediatrician and hospital executive board member, a professor of medical informatics, an entrepreneur with a background in open-source technology, a medical doctor and high-risk patient, a senior doctor in hygiene, a digital health connector, a designer, a chief information and digital officer, and the managing director of a research institution.

During the final presentations, teams were asked to first name the challenge or problem intended to be solved, then showcase the solution developed, and how it can be integrated into health care systems. The winning teams were awarded monetary prizes of 5000 EUR (US $6059) in total following the evaluation criteria shown in Table 2.

**Table 2:** Evaluation criteria for the projects presented in the final pitch session at the end of the hackathon.

Criterion	Considerations
Innovation	How innovative is the idea? Does the approach stand out from solutions that are available so far?
Impact	How high is the benefit for the patient and other stakeholders of the health care system? Does the solution generate impact for our society? How viable is the idea?
Applicability	How user-friendly is the solution? Is the solution convenient to use? How viable is the solution?
Feasibility	How realistic is the implementation of the idea? Can the solution be brought to life by further research projects or by founding a company?
Execution	How sophisticated and well-made is the technical implementation?

### Data Collection and Analysis

Two online surveys were designed by the Hacking Health Berlin team, one survey targeting participants and another one for mentors of the hackathon (Multimedia Appendices 1 and 2). The research team members comprised health care professionals, scientists, and entrepreneurs with backgrounds in anesthesiology, intensive care medicine, pediatrics, psychology, microbiology, medical informatics, neuroscience, human factors, health care building design, human-centered design, and marketing. Google Forms was used to collect responses. CHERRIES (Checklist for Reporting Results of Internet E-Surveys) was used to guide survey development (Multimedia Appendix 3) [56].

The participant survey comprised 17 plus 5 optional items (using branching logic, dependent on responses to previous questions). The questions assessed quality experience throughout the online hackathon, including the mentorship sessions and future plans regarding the projects presented. Furthermore, mentors provided feedback to the organizers via an online survey through Mentornity subsequently to each session (Multimedia Appendix 1). All data were collected during a single event with surveys being distributed to mentors and participants at the end of the hackathon. Surveys were anonymous and voluntary and included electronic consent. Participant responses were collected, managed, and analyzed using Google Forms. A thematic hackathon T-shirt was offered as a reward for completing the surveys.

## Results

### Participants

In total, 171 participants, of whom 42% (n=72) were female, expressed their interest and preregistered online within 5 days prior to the hackathon. Of the total, 84 participants had joined the Zoom event for the opening ceremony, 62 of whom formed 23 different teams. Finally, 14 teams comprising 48 members pitched their completed projects at the end of the hackathon.

Participants based in at least 8 countries participated, including Germany (n=25, 52%), the United States (n=5, 10%), Bosnia and Herzegovina (n=3, 6%), Canada (n=3, 6%), and others (n=5, 10%) like Belgium, Sri Lanka, Switzerland, and the United Kingdom, as well as 7 participants (15%) from unknown locations. Various professional backgrounds were represented, with IT (n=8, 17%), research (n=5, 10%), project management (n=4, 8%), and software engineering (n=4, 8%) being the most frequent work fields. Other backgrounds (n=27, 56%) included business development, data science, entrepreneurship, health care, human resource management, mechanical engineering, quality management, education, and UI/UX (user interface/user experience) design. Half of the participants (n=24) had never joined a hackathon before.

### Mentoring

A total of 60 mentors preregistered online over a period of 6 days prior to the hackathon. Of them, 52 completed their profiles and became active on the Mentornity platform, of whom 46 joined Slack to communicate with teams and organizers. A briefing session was held to inform mentors about the online hackathon concept and how to offer expertise and express their availability to participants. In total, 75 video mentoring sessions took place on Mentornity where 35 mentors and 19 teams interacted. The actual number of video sessions is estimated to have been higher since it was known that other video conference platforms such as Zoom, Slack, and Skype were used and not tracked by Mentornity.

**Figure 2:**
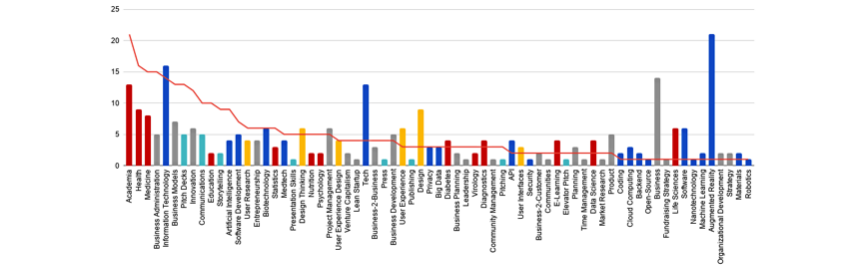
Sessions booked in the different fields of expertise available for mentorship (red line) and the number of mentors with knowledge in the respective disciplines (bars). Participants booked mentoring sessions in the field of tech (blue bars); health care, research, and education (red bars); entrepreneurship and management (gray bars); public speaking and public relations (teal bars); user research and design (yellow bars). API: application programming interface.

Of all mentors, 24 had no previous experience mentoring, 12 had experience from a previous Hacking Health Berlin hackathon, and the remaining 16 had experience mentoring but were new to Hacking Health Berlin events. The group of mentors had broad professional backgrounds as shown in Figure 2. The top fields of expertise comprised business, medicine, and software development. Along those lines, the most booked mentoring sessions reflected a high interest in these fields.

Feedback forms (Multimedia Appendix 1) were automatically created on Mentornity for each mentoring session conducted. Of the mentors, 13 completed the feedback forms, covering a total of 25 mentoring sessions involving 13 teams. The feedback showed that mentors were largely satisfied with the online mentoring experience despite the new format. Of 25 mentoring sessions, 22 took place and were evaluated with ratings ranging from 3 to 5, scoring an average of 4.68 (SD 0.57). Three surveys indicated that the mentoring session did not occur for various reasons: cancellation of the meeting, difficulties with the platform, or the teams did not attend the scheduled mentoring session. The teams that received mentoring reported gaining valuable insights, although they also highlighted that online mentoring was a new format and brought interaction and scheduling challenges. A number of positive comments by mentored teams reflected appreciation for mentors’ willingness to share their experience, which enabled them to take a broader perspective of their project. However, during the hackathon, some teams and mentors reported difficulties in scheduling sessions or connecting via the platform, resulting in some frustration.

### Projects

A total of 14 projects were developed and presented to the jury and public audience. The scope of the created solutions included telemedicine services, personal risk assessment tools, trackers for COVID-19–specific symptoms and mental health impairments, tracers for population density and risk contacts, immunity passports, hardware for safe reuse of personal protective equipment, platforms for sharing food and other essential products, and educational tools.

The *most innovative* solution [57] consisted of a mobile app for knowledge transfer between researchers, the public, and health care workers during and after the pandemic. It tackled the need to access clear and timely COVID-19 scientific evidence for researchers and practitioners to have a clear picture of the situation. App users were able to share information through short videos and visual graphics, aided by curated information and deep learning. The team presented a functional website presenting an novel artificial intelligence–based tool where users could enter specifics about their professional background and their individual research question in a free-text field to obtain summarized research findings from peer-reviewed scientific literature.

The *most impactful* solution [58] was a service platform with a video-based hotline to provide instant psychological support for health care professionals exposed to stress, a high workload, and moral dilemmas during the pandemic. With restricted time, health care workers lack the capacity to reach out to existing psychosocial support structures. By bringing psychological support to health care professionals directly via a ​video chat hotline, the instant psychological support was made possible by the voluntary commitment of a broad network of psychotherapists. With a well-planned concept presented on a functional website, the project straightforwardly addressed an imminent user need as identified by frontline health care professionals and outlined in challenge 5.

The *most applicable* solution [59] consisted of an app that allowed frontline health care workers involved in COVID-19 patient care to perform regular virtual health self-checks and seek support to ensure their mental, as well as physical health, was prioritized. The presented mock-up of the app had a simple user interface, self-checks required very little time, and support tools were easy to access.

The app rated as *most feasible* [60] helped to redistribute critical resources and services. As the pandemic has disrupted supply chains around the world, at the time plenty of resources available were not arriving at locations where needed. The platform solved the issue by allowing people to easily know what was needed, indicate where to send these resources, and track the resource shipping progress. A web portal ready for immediate implementation and use, as well as an outline of short and long-term goals of the project, was presented.

The *best executed* solution [61] focused on privacy-first contact tracing and digital epidemiology. It created a multiservice platform connecting patients to self-assessment tools, dashboards, and personal analytics. Users would be able to easily track their logins on their mobile phones and see how symptoms changed. The secure and anonymous contact tracing would allow patients to be aware in their residential area and avoid potential high-risk areas. Finally, information like location, age, and test results would be collected and anonymized into a large database for further analyses and data modeling. The team presented a working web app prototype with 3 features (self-assessment for tracking COVID-19–specific symptoms, contact tracing, warning function for population-dense areas) with a user-friendly and professional-looking user interface, as well as a clear roadmap for the future implementation of their product.

Approaches by other teams included sterilization methods to reuse protective equipment for health care workers, virtual reality visualizations of epidemiological data, and tools to support social distancing in public spaces. The results presented varied from concepts and click dummies to working prototypes of mobile apps.

### Hackathon Experience

According to the feedback (n=30), participants were made aware of the event by word of mouth (n=14, 47%), social media (n=12, 40%), event platforms (n=3, 10%), email, and postings on Hacking Health Berlin’s and Data Natives’ existing online communication channels on Slack (n=4, 13%).

**Figure 3:**
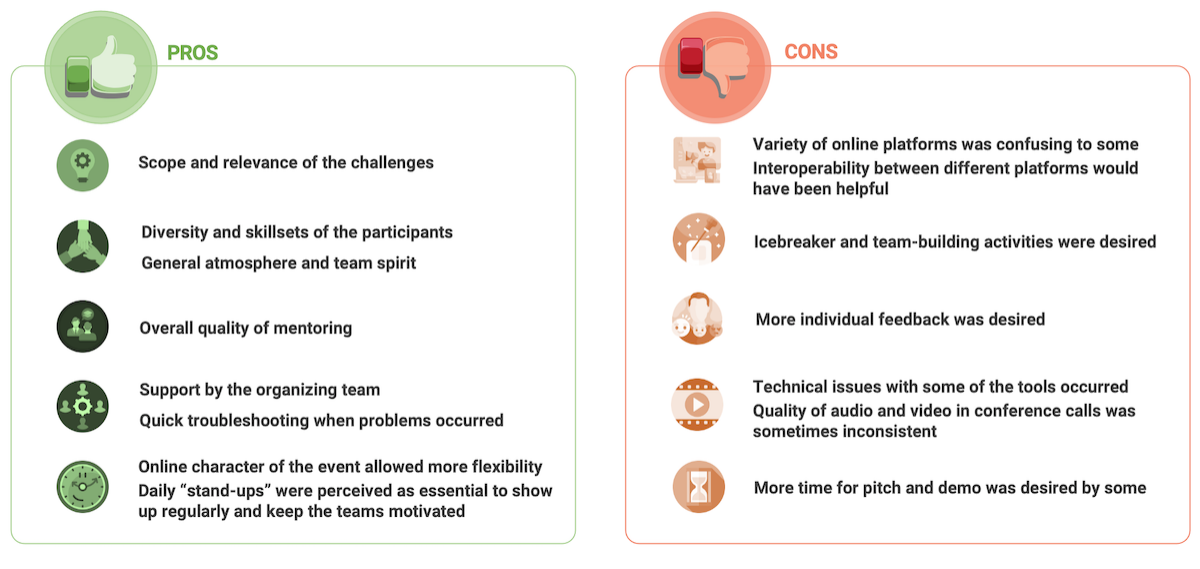
Experiences of participants specifically regarding the remote and online nature of the hackathon.

Participants were overall satisfied with the execution of the remote hackathon, evaluating the experience with 4.23 points out of 5 on average. They appreciated the general atmosphere and team spirit, diversity and skillsets of the participants and mentors, the flexibility permitted by the remote nature of the event, and felt that occurring problems could be quickly resolved by the organizing team. However, the variety of online platforms that were used was perceived to be overwhelming to some, and technical issues with the tools were reported. Furthermore, icebreaker and team-building activities at the beginning of the event were desired to get familiar with the general concept of online hackathons and to get to know the other participants (Figure 3). Most participants (n=21, 70%) felt that it was an advantage that the hackathon was held over a long holiday weekend, but opinions were mixed about whether an in-person event of a longer duration than the usual 48 hours would be desirable, with 40% (n=12) in favor of the idea.

Before the hackathon, participants rated their confidence in starting a digital health venture or research project with 2.6 points out of 5 on average. After the hackathon, the participants’ confidence increased significantly to 3.7 on average. Of the participants, 60% (n=18) would not have started their projects without attending this hackathon, and agreed that it enabled them to progress faster by (1) offering new insights and feedback provided by the mentors, (2) creating a functional prototype, and (3) finding the right members for their team.

## Discussion

### Main Findings

This case study demonstrates the feasibility and effectiveness of remote hackathons as an approach to combat the challenges of the COVID-19 pandemic. The event was prepared in a much shorter time and conducted entirely online at significantly lower costs, with similar results as health hackathons held in person. To our knowledge, this is one of the first studies to report and critically discuss experiences of, and provide guidance on, online hackathons and hackathons specifically targeted to solve the challenges around COVID-19, as well as to report on the impact of expert mentoring during health hackathons.

### Cultural Change and Opportunities of Online Collaborations

The circumstances of the COVID-19 pandemic and the necessity for social distancing have led to a cultural change in work settings, communication, and collaboration. As a consequence, professional meetings and conferences are more likely to take place in an online setting. Based on the high number of participants and mentors, the medical and scientific way in which the challenges were addressed, and the feedback from participants who had experience in face-to-face hackathons, we can conclude that remote online hackathons are feasible and resemble some of the dynamics and synergies of the in-person ones. Online hackathons effectively bring people together at a significantly lower cost in comparison to a conventional in-person hackathon. The EasterHack event enabled participants not only to collaborate in an interdisciplinary manner but also across diverse geographic locations, avoiding the possible inconveniences and costs of travel. The transition toward an online and federated event allowed for a more diverse and global audience. There is substantial research showing a relation between increased diversity in teamwork leads and benefits, such as increased creativity and better problem-solving abilities [50,51].

Since the first online hackathons to fight COVID-19 were launched in March 2020, more than 50 online events summing up to 28,000 participants [62-67] have been held. The impact is evident in terms of a higher participation level and number of collaborating partners, which brings a multibeneficial bottom line of fewer logistical, financial, geographical, and time constraints, and reduced carbon dioxide output.

### The Impact of Expert Mentoring

In recent years, mentoring has been shown to have a positive impact on the way participants gain insights, interact, execute multidisciplinary tasks, and build their prototypes during hackathons [3,6,8,28,36]. They act as guiding lights during the process by encouraging exploration and providing information in areas where participants have questions and interests. The main benefit is reflected in the holistic and complementary nature of each hack, often embracing different disciplines and resulting in outstanding solutions and pitches with a strong technical background (software or hardware); addressing health-related challenges pragmatically; and putting the whole idea into a well-polished business plan. On those grounds, the recruitment of expert mentors takes place depending on the scope of the hackathon.

In general, challenges tackling the COVID-19 pandemic are complex and require critical advice from biomedical experts and frontline health care professionals. These hurdles can be tackled through expert mentorship, complemented with knowledge from specialists for additional areas of expertise aiming to increase the feasibility and applicability of solutions. Availability of mentors is important; for example, health care staff and other essential workers may have commitments directly related to the pandemic, which limits their availability. With online mentoring programs, mentors can help participants with flexible and short mentorship sessions without altering their working schedules and personal time. Likewise, mentors located in different regions and time zones were able to support participants, which in an in-person format would not have been the case. The interdisciplinary nature and outcome of the event was reflected in the diversity of sessions booked; this also confirms the relevance of mentoring and our endeavor to have medical expertise as the backbone. For these reasons, we strongly recommend online mentorship to be considered in both virtual and on-site hackathons.

### Challenges of Conducting Hackathons Remotely

Online hackathons might have some limitations such as data security concerns when it comes to personal information, especially with video conference tools, communication breakdowns, technical issues, and access to essential hardware including microphones and cameras. Only recently, the video conference service Zoom reported security issues concerning unwanted guests who disturbed some virtual meeting rooms [67-69]. While the preparation time for online hackathons can be relatively short, all tools and technical solutions need to be tested thoroughly in advance to avoid frustration among organizers, participants, and mentors. Technical glitches may also affect the ability to communicate properly, share announcements, recruit survey participants, and marketing. Furthermore, participants who work remotely might become distracted more easily with the screen being the only visual feedback platform and other work-related and personal commitments at the same time. Therefore, a certain level of media competence is recommended. Online team building activities require different approaches compared to events in physical presence. In addition, the dynamics of networking and small talk might be different. Although participants of EasterHack reported positive experiences regarding group synergies and team spirit during the event, there were 14 individuals who did not complete their projects. Further research should address drop-out reasons and challenges of attending online hackathons from the participant perspective, and elaborate on how these challenges might be resolved.

### Limitations

This case study was based on data that were captured in a single online event and not directly compared with in-person events. Although participants were from various geographical locations, the sample size of 48 participants limits broad generalizations. Further studies in the course of online hackathons should be conducted to validate the stated findings.

### Conclusions

Online hackathons offer an ideal setting for rapid and goal-oriented collaborative work, and a format to react quickly to urgent and unknown situations like the COVID-19 pandemic.

EasterHack participants generally reported a positive experience during the online hackathon format, increased confidence, and a higher likelihood of continuing to work on their projects. Despite remotely held events posing challenges to organizers, participants, and mentors, they can be executed much faster and at lower costs compared to in-person meetings, as well as foster team diversity and increase cross-regional collaboration. Setting up a research project and creating IT solutions within health care or research facilities can be a time-consuming and onerous process; however, concept-to-solution processes can be achieved during a single online hackathon weekend. As highlighted by an avid supporter of hackathons [70]:

Governments need to change. Institutions need to change. But, in the meantime, we have hackathons, where you can rapidly prototype. [...] Do you get a perfect solution? No. But anything is better than nothing. Done is better than perfect.

## Appendix

Multimedia Appendix 1 Postsession feedback form for mentors to respond to on the Mentornity platform.

Multimedia Appendix 2 Feedback survey for hackathon participants.

Multimedia Appendix 3 Checklist for Reporting Results of Internet E-Surveys (CHERRIES).

## Abbreviations

CHERRIES: Checklist for Reporting Results of Internet E-Surveys
IT: information technology
UI: user interface
UX: user experience
