# Supplementary material for: Interdisciplinary Online Hackathons as an Approach to Combat the COVID-19 Pandemic: Case Study
Source: J Med Internet Res. 2021 Feb 8;23(2):e25283. doi: 10.2196/25283 (PMC7872325; doi:10.2196/25283)
Supplement: Multimedia Appendix 1 [file jmir_v23i2e25283_app1.docx]

**Multimedia Appendix 1: Postsession feedback form for mentors to respond to on the *Mentornity* platform.**

1. How was your mentoring session? Do you feel that this mentoring session has increased the quality, feasibility, and impact of the project? *(free-text answer)*
2. Rate your mentoring session. How satisfied are you with the team's response to your suggestions? *(Likert scale from 1 to 5, with 1 being the lowest score)*
3. Was it easy to communicate about scheduling the session with the team? *(yes/no)*
4. Did you gather new insights during the session? *(free-text answer)*
5. How would you evaluate your experience in an online-only hackathon vs. an in-person event? *(free-text answer)*
