# Supplementary material for: Interdisciplinary Online Hackathons as an Approach to Combat the COVID-19 Pandemic: Case Study
Source: J Med Internet Res. 2021 Feb 8;23(2):e25283. doi: 10.2196/25283 (PMC7872325; doi:10.2196/25283)
Supplement: Multimedia Appendix 2 [file jmir_v23i2e25283_app2.docx]

**Multimedia Appendix 2: Feedback survey for hackathon participants.**

1. Was this the first hackathon you have ever attended? *(yes/no)*
   - If yes: Have you ever participated in an online/remote hackathon? *(yes/no)*
     1. If yes: If this was not your first remote/online hackathon: What did you like and dislike compared to other online/remote hackathons? *(free-text answer)*
   - If no: If you haven't participated in a remote/online hackathon: What did you like and dislike compared to in-person hackathons? *(free-text answer)*
2. How did you like the setup and structure of the hackathon? *(Likert scale from 1 to 5, with 1 being the lowest score)*
3. What did you like about this hackathon? *(free-text answer)*
4. What did you not like about this hackathon? *(free-text answer)*
5. What aspects of an in-person hackathon did you most miss in an online/remote hackathon? *(checkboxes)*
   - Physical presence of organizers
   - Physical presence of the team
   - Physical presence of mentors
   - Physical activities (such as yoga, workshops etc.)
   - Keynotes and panel events
   - Food
   - Swag (t-shirts, bags, etc.)
   - Nothing at all
6. Were the daily standups a useful substitute for in-person meetings? How did you feel about the frequency? Did you feel like your needs and questions were met by the organizing team? *(free-text answer)*
7. If you were to attend another online/remote hackathon, what would you improve upon? *(free-text answer)*
8. The EasterHack hackathon ran for 4 days over a holiday weekend. Do you feel that this was an advantage or a disadvantage? *(multiple choice)*
   - Definitely advantage
   - More of an advantage
   - Did not matter
   - More of a disadvantage
   - Definitely disadvantage
9. Do you think a longer hackathon would be an advantage or a disadvantage for an in-person hackathon? *(multiple choice)*
   - Definitely advantage
   - More of an advantage
   - Did not matter
   - More of a disadvantage
   - Definitely disadvantage
10. During the EasterHack, we had some additional sessions such as mentoring, coaching, and meditations. What insights or benefits did you gain from these? *(free-text answer)*
11. How helpful were the sessions with the mentors? *(Likert scale from 1 to 5, with 1 being the lowest score)*
12. In what areas did you gain new insights during the mentor and coaching sessions? *(checkboxes)*
13. How would you rate your confidence to start your own venture in healthcare, or digital health research project before the hackathon? *(Likert scale from 1 to 5, with 1 being the lowest score)*
14. How would you rate your confidence to start your own venture in healthcare, or digital health research project after the hackathon? *(Likert scale from 1 to 5, with 1 being the lowest score)*
15. How likely is it that you will keep working on the idea you have developed during the Hackathon? *(Likert scale from 1 to 5, with 1 being the lowest score)*
16. Would you have started working on your project without this hackathon? *(free-text)*
    - If yes: Has the hackathon enabled you to progress faster? And how? *(free-text answer)*
    - If no: Why not? *(free-text answer)*
17. How did you hear about the EasterHack Hackathon? *(checkboxes)*
