# Supplementary material for: Interdisciplinary Online Hackathons as an Approach to Combat the COVID-19 Pandemic: Case Study
Source: J Med Internet Res. 2021 Feb 8;23(2):e25283. doi: 10.2196/25283 (PMC7872325; doi:10.2196/25283)
Supplement: Multimedia Appendix 3 [file jmir_v23i2e25283_app3.docx]

**Appendix 3:** **Checklist for Reporting Results of Internet E-Surveys (CHERRIES).**

| **Category** | **Checklist item** | **Explanation** | **Response** |
| --- | --- | --- | --- |
| **Study Design** | Describe Survey Design | Describe target population, sample frame. Is the sample a convenience sample? | The target population of the survey is being described in the Methods section of the article as participants and mentors of the “EasterHack - #HackTheCurve” online remote hackathon. |
| **IRB** | IRB approval | Mention whether the study has been approved by an IRB | As an anonymous survey, this does not apply. |
|  | Informed Consent | Where were the participants told the length of time of the survey, which data were stored & where & for how long, the investigator and purpose of the study? | The survey contained study information and an informed consent form. |
|  | Data protection | If any personal information was collected or stored, describe what mechanisms were used to protect unauthorized access. | The survey was anonymous. No personal information was collected unless a t-shirt was ordered (name, residential address); however, this data was not brought into context with the survey data. Only the research team has access to the collected data. |
| **Development and pre-testing** | Development and testing | State how the survey was developed, including whether the usability and technical functionality of the electronic questionnaire had been tested before fielding the questionnaire | The survey was designed by the interdisciplinary research team, created and tested using Google Forms. |
| **Recruitment process and description of the sample having access to the questionnaire** | Open survey versus closed survey | An “open survey” is a survey open for each visitor of a site, while a closed survey is only open to a sample which the investigator knows (password-protected survey) | Access links were only provided to a closed online chat (Slack) with participants and mentors of the hackathon. |
|  | Contact mode | Indicate whether or not the initial contact with the potential participants was made on the internet. (Investigators may also send out questionnaires by mail and allow for Web-based data entry.) | Access links were provided at the end of the event when previous communication was already established between the organizing team and participants/mentors. |
|  | Advertising the survey | How/where was the survey announced or advertised? Some examples are offline media (newspapers), or online (mailing lists - if yes, which ones?) or banner ads (Where were these banner ads posted and what did they look like?. It is important to know the wording of the announcement as it will heavily influence who chooses to participate. Ideally, the survey announcement should be published as an appendix. | Access links were published in a closed online chat (Slack) only available to participants and mentors of the hackathon. |
| **Survey Administration** | Web/E-Mail | “state the type of e-survey (eg one posted on a web site or one sent out through e-mail). If it is an e-mail survey, were the responses entered manually into a database, or was there an automatic method for capturing responses?” | Responses were automatically captured in Google Forms. |
|  | Context | Describe the Web site (for mailing list/ newsgroup) in which the survey was posted. What is the Website about, who is visiting it, what are visitors normally looking for? | Closed group chat (Slack) used for the purpose of updates and internal communication during the hackathon. |
|  | Mandatory/voluntary | Was it a mandatory survey to be filled in by every visitor who wanted to enter the Web site, or was it a voluntary survey? | Participation was anonymous and voluntary. |
|  | Incentives | Were any incentives offered (eg. monetary prizes, or non-monetary incentives such as an offer to provide the survey results)? | Participants could choose to order a hackathon themed t-shirt at the end of the survey free of charge. |
|  | Time/Date | In what timeframe were the data collected? | Data was collected from 13th - 20th April 2020. |
|  | Randomization of items or questionnaires | To prevent biases items can be randomized or alternated | The survey items were not randomized or alternated. |
|  | Adaptive questioning | Use adaptive questioning (certain items, or only conditionally displayed based on response to other items) to reduce number and complexity of the questions) | Adaptive questioning was used, e.g. when participants indicated they have previously participated in, or mentored in a previous hackathon. |
|  | Number of Items | What was the number of questionnaire items per page? The number of items is an important factor for the completion rate. | The survey comprised of 22 total items, of which 17 were posed to all participants, and 5 questions were optional, depending on branching logic. |
|  | Number of screens (pages) | Over how many pages was the questionnaire distributed? The number of items is an important factor for the completion rate. | The survey was distributed on a single page. |
|  | Completeness check | It is technically possible to do consistency or completeness checks before the questionnaire is submitted. Was this done, and if “yes”, how (usually JAVAScript)? An alternative is to check for completeness after the questionnaire has been submitted (and highlight mandatory items). If this has been done, it should be reported. All items should provide a non-response option such as “non-applicable” or “rather not say” , and selection of one response option should be enforced | We used the built-in completeness check of Google Forms for all mandatory questions. |
|  | Review Step | State whether respondents were able to review and change their answers (eg through a Back button or a Review step which displays a summary of the responses and asks the respondents if they are correct). | Participants were able to review and change their answers before the final submission. |
| **Response rates** | Unique site visitor | If you provide view rates or participation rates, you need to define how you determined a unique visitor. There are different techniques available, based on IP-addresses or cookies or both. | Not available. |
|  | View rate  (Ratio of unique visitors who agreed to participate/unique first survey page visitors) |  | Not available. |
|  | Participation rate  (Ratio of unique visitors who agreed to participate/ unique first survey page visitors) |  | The participation rate was 100%. |
|  | Completion rate  (Ratio of user who finished the survey/ users who agreed to participate) |  | The completion rate was 100%. |
| **Preventing multiple entries from the same individual** | Cookies used | Indicate whether cookies were used to assign a unique user identifier to each  client computer. If so, mention the page on which the cookie was set and read,  and how long the cookie was valid. Were duplicate entries avoided by preventing  users access to the survey twice; or were duplicate database entries having the  same user ID eliminated before analysis? In the latter case, which entries were  kept for analysis (eg, the first entry or the most recent)? | Pre-built-in cookies were used on Google Forms. |
|  | IP check | Indicate whether the IP address of the client computer was used to identify potential  duplicate entries from the same user. If so, mention the period of time  for which no two entries from the same IP address were allowed (eg, 24 hours).  Were duplicate entries avoided by preventing users with the same IP address  access to the survey twice; or were duplicate database entries having the same  IP address within a given period of time eliminated before analysis? If the latter,  which entries were kept for analysis (eg, the first entry or the most recent)? | No IP addresses were collected. The results were manually checked for duplications. |
|  | Log file analysis | Indicate whether other techniques to analyze the log file for identification of  multiple entries were used. If so, please describe. | Not applicable. |
|  | Registration | In “closed” (non-open) surveys, users need to login first and it is easier to prevent  duplicate entries from the same user. Describe how this was done. For example,  was the survey never displayed a second time once the user had filled it in, or  was the username stored together with the survey results and later eliminated?  If the latter, which entries were kept for analysis (eg, the first entry or the most  recent)? | Does not apply. |
| **Analysis** | Handling of incomplete questionnaires | Were only completed questionnaires analyzed? Were questionnaires which terminated  early (where, for example, users did not go through all questionnaire  pages) also analyzed? | No incomplete questionnaires were submitted. |
|  | Questionnaires submitted with an atypical timestamp | Some investigators may measure the time people needed to fill in a questionnaire and exclude questionnaires that were submitted too soon. | Does not apply. |
|  | Statistical correction | Indicate whether any methods such as weighting of items or propensity scores  have been used to adjust for the non-representative sample; if so, please describe  the methods. | Does not apply. |
